# Cloning and bioinformatics analysis of key gene *ShOMT3* of podophyllotoxin biosynthesis pathway in *Sinopodophyllum hexandrum*

**DOI:** 10.1371/journal.pone.0314919

**Published:** 2025-02-14

**Authors:** Wei Liu, Haona Gao, Dan Zhao, Shuying Li, Lu Li, Xiufang Zhao, Zheng Zhang, Dongxue Yin

**Affiliations:** 1 College of Agriculture, Henan University of Science and Technology, Luoyang, China; 2 Traditional Chinese Medicine (ZhongJing) School, Henan University of Chinese Medicine, Zhengzhou, China; 3 College of Agricultural Equipment Engineering, Henan University of Science and Technology, Luoyang, China; Fujian Provincial Hospital, CHINA

## Abstract

*Sinopodophyllum hexandrum* (*S. hexandrum*) is an endangered traditional Chinese medicine as abundant podophyllotoxin with powerful anticancer activity. In this study, the rootstalks of *S. hexandrum* from different geographical locations in China [S1 (Gansu) and S2 (Shaanxi)] were used as research materials to clone the key gene pluviatolide O-methyltransferase 3 (*ShOMT3*) in the podophyllotoxin biosynthetic pathway. Subsequently, bioinformatics analysis of the *ShOMT3* gene and its encoded protein was subjected to bioinformatics analysis using various analysis software including ProtParam, DeepTMHMM, SubLoc, Signal-P 5.0, and Swiss-model. The results of the analysis revealed that the CDS region of the *ShOMT3* gene is 1119 bp long, encoding 372 amino acids. The theoretical molecular weight of the *ShOMT3* protein is 41.32784 kD, and the theoretical isoelectric point (pI) is 5.27. The instability coefficient of the protein is 46.05, the aliphatic index is 93.58, and the grand average of hydropathicity (GRAVY) is 0.037, indicating that it is an unstable hydrophobic protein. The protein does not contain transmembrane domains or signal peptides, indicating that it is a non-secreted protein. Secondary structure prediction results suggests that the protein consists of alpha helices, random coils, extended strands, and beta-turns. Tertiary structure prediction results suggests that the protein functions as a monomer. In the phylogenetic tree, the *ShOMT3* protein has the highest homology with *Podophyllum peltatum* (*P. peltatum*). The successful cloning and bioinformatics analysis of the *ShOMT3* gene provide theoretical basis and excellent genetic resources for the molecular regulatory mechanism analysis of the podophyllotoxin biosynthetic pathway and molecular breeding in *S. hexandrum*.

## Introduction

*S. hexandrum*, a perennial herb belonging to the *Berberidaceae* family, is a traditional Chinese medicinal material [1]. It is primarily distributed in Southeast Asia, thriving in alpine grasslands and forest edges at altitudes ranging from 1300 to 4500 meters [2]. This herb has long been used in traditional Chinese medicine for various therapeutic purposes, making it a valuable addition to the pantheon of Chinese medicinal plants [3]. Podophyllotoxin, a primary medicinal component of *S. hexandrum*, exhibits a diverse array of biological activities including cancer cell growth inhibition, antiviral activity, and insecticidal properties [4–7]. After rigorous clinical research using podophyllotoxin and its derivatives on various cancers such as leukemia, prostate cancer, cervical cancer, thymoma, and glioblastoma multiforme, it was found that podophyllotoxin exhibits broad-spectrum and highly efficient anticancer activity [6,8,9]. Subsequently, podophyllotoxin-based podophyllin antitumor drugs were continuously developed [10]. The first batch of podophyllin antitumor drugs, such as etopophos, etoposide (VP-16) and teniposide (VM-26), were approved for marketing in Switzerland and the United States in the 1980 [11]. Due to the further development of novel epipodophyllotoxin antineoplastic drugs in the fields of medicine, biology, chemistry, and beyond, the demand for podophyllotoxin, its precursor substance podophyllotoxin, in the international market has been increasing [12,13]. Currently, the natural source of podophyllotoxin is primarily derived from *S. hexandrum* and some other *Epipodophyllaceae* plants [3,14].*S. hexandrum* The growth of *S. hexandrum* is closely associated with various ecological factors such as altitude, temperature, and rainfall [15,16]. It is typically found in remote and high-altitude regions that are characterized by cold temperatures, including Tibet, Yunnan, Sichuan, and Qinghai provinces in China [17]. In recent years, environmental damage has caused a reduction in the suitable habitat range of *S. hexandrum*, while market demand and economic interests have driven humans to overexploit this species through excessive harvesting [3,18]. This has led to a growing scarcity and rarity of *S. hexandrum*’s wild resources. Extracting podophyllotoxin from plants results in low extraction rates and causes significant damage to wild plant resources, making it unsustainable [19]. Chemical synthesis methods, due to their cumbersome procedures, high costs, and slow production rates, are unsuitable for large-scale production of podophyllotoxin and its derivatives [20]. In contrast, the emergence of Biosynthesis methods has provided a novel and reliable approach for the efficient synthesis of significant amounts of podophyllotoxin, thus fulfilling the commercial demand [21,22].

Methyltransferases (MTs) are widely present in nature and participate in various important biological processes, including signal transduction, transcriptional regulation, and biosynthesis of metabolic products [23]. Based on the type of methyl acceptor, MTs can be classified as oxygen-, nitrogen-, carbon-, sulfur-, and other methyltransferases [24]. Among them, oxygen methyltransferases (OMTs) account for 54% of MTs and usually use S-adenosylmethionine as the methyl donor [25]. In animals, O-methylation plays a significant role not only in the detoxification of reactive hydroxyl groups but also in the biosynthesis of hormone melatonin and catecholamine neurotransmitters [26]. OMTs in plants typically act on compounds containing phenolic hydroxyl groups, such as phenylpropanoids, flavonoids, and alkaloids, to methylate their oxygen atoms [27,28]. They are a crucial regulatory enzyme in the phenylpropanoid metabolic pathway, which is derived from the shikimate pathway [29]. Pinane, the common precursor of podophyllotoxin, is one of the important natural secondary metabolites in the phenylpropanoid metabolic pathway of plants [30]. Previous research has demonstrated that caffeic acid 3-O-methyltransferase (COMT) and caffeoyl-CoA O-methyltransferase (CCoMT), two types of OMTs, play a critical regulatory role in the biosynthesis pathway of pinnae [31,32]. In previous studies, our team conducted transcriptome analysis of *S. hexandrum* from three different provenances in Gansu and Shaanxi. The results indicated significant differences in the expression of the Cluster-2923.2731 gene. Upon alignment, it was found that the Cluster-2923.2731 gene had a consistent sequence with the KT390157.1 and MW531736.1 genes in the NCBI database that encode OMT3 protein in *S. hexandrum*. In this study, *ShOMT3* was identified as an important regulatory enzyme gene involved in the downstream synthesis of podophyllotoxin from pluviatolide to deoxypodophyllotoxin in *S. hexandrum*. The regulatory role of this enzyme gene in the podophyllotoxin biosynthesis pathway has been characterized [33,34]. Lau and Sattely [35] conducted a transcriptome analysis of *P. hexandrum* in the database and identified several candidate enzymes involved in the downstream biosynthesis of podophyllotoxin: O-methyltransferases (OMT1, OMT2, OMT3, OMT4), CYPs, and a 2-oxopentanedioic acid/Fe(II)-dependent dioxygenase (2-ODD). These enzymes were co-expressed with CYP719A23 in tobacco leaves, and it was found that only *ShOMT3* could catalyze the C-4′ hydroxylation of (−)-pluviatolide to produce (−)-5′-desmethoxy-yatein [34,36].

Currently, the biosynthetic pathways for important intermediates, namely deoxypodophyllotoxin and 4’-desmethyl-epipodophyllotoxin, have been well characterized [37]. However, the key enzymes involved in the downstream process of podophyllotoxin biosynthesis have not been definitively identified. Domestic and international scholars have conducted extensive research on the pharmacological effects, derivative synthesis, and biosynthetic pathway of podophyllotoxin [6,38]. However, the understanding of the critical regulatory genes involved in its biosynthesis is not yet fully developed [39]. Moreover, most of the existing references on plant oOMTs have focused on the study of COMT, which regulates lignin biosynthesis [27,40]. However, the research on the *ShOMT3* gene in the podophyllotoxin biosynthetic pathway of *S. hexandrum* is relatively limited. Therefore, in this study, the *ShOMT3* gene from samples S1 and S2 was cloned and comprehensively bioinformatics analyzed. The research findings contribute to the understanding of the molecular mechanism of podophyllotoxin biosynthesis in *S. hexandrum*, and have important theoretical and practical implications for the industrial production of podophyllotoxin and the molecular breeding of *S. hexandrum*.

## Materials and methods

### Plant materials

The test materials were collected from the *S. hexandrum* germplasm resource nursery at Henan University of Science and Technology (E112°25’12”, N34°37′12.6″). This nursery was established in August 2019, and the *S. hexandrum* seedlings were introduced from various locations including Dalagou in Gansu Province’s Diebu County, Ming Xing Temple in Shaanxi Province’s Mount Taibai, Bayi Town in Tibet’s Nyingchi, and Shangri-la in Yunnan Province. After three years of growth in the nursery, the test materials were collected in June, 2022. The roots of *S. hexandrum* from Gansu (S1) and Shaanxi (S2) were collected as the test materials. The roots were cleaned with distilled water to remove the soil, rinsed with TE buffer, and then quickly frozen in liquid nitrogen. They were stored at -80°C in a refrigerator for subsequent experiments.

### Extraction and cDNA synthesis of *S. hexandrum*

RNA was extracted from *S. hexandrum* using the RNArep Pure Polysaccharide Polyphenol Plant Total RNA Extraction Kit (TianGen Biotech, Beijing) according to the manufacturer’s instructions. RNA integrity was assessed using 1% agarose gel electrophoresis, and RNA concentration and purity were determined using the Nanodrop 2000 Ultra-Micro Spectrophotometer. Following the manufacturer’s instructions for the FastKing cDNA First Strand Synthesis Kit (TianGen Biotec, Beijing), cDNA first strand was synthesized by reverse transcription. The synthesized cDNA was then stored at -80°C in the refrigerator for future use.

### Cloning of *ShOMT3* gene

Using the *ShOMT3* gene sequence published on NCBI (GenBank: MW531736.1) as a reference, primers were designed for the CDS (coding sequence) of the gene, with the forward primer *ShOMT3*-F: 5’-ATGGAAATGGCTCCAACAATG-3’ and the reverse primer *ShOMT3*-R: 5’-TTAGGGAAACACTTCAATGATAGACTT-3’. PCR amplification was carried out using cDNA obtained through reverse transcription as a template, with TransStart FastPfu DNA Polymerase (TransGen Biotech, Beijing) as the enzyme. Set up the PCR amplification reaction with an initial denaturation at 95 °C for 3 mins, followed by 32 cycles of denaturation at 94 °C for 25 seconds, annealing at 56 °C for 25 s, and extension at 72 °C for 10-15 s/kb. After completing the cycles, conduct a final extension at 72 °C for 5 minutes, and then store the reaction at 4 °C until further use. Due to the inability of this enzyme to add an A base to the end of the product, the PCR product was purified using the M5 Gel Extraction Kit (with column) (Mei5 Biotechnology, Beijing) for gel extraction. The purified target gene fragment was then A-tailed using the DNA A-Tailing Kit (Takara Biomedical Technology, Beijing) to add a ‘A’ tail at the 3’ end for ligation with the pMD18-T vector. After transforming E. coli DH5α competent cells, positive clones were screened using 2 × M5 HiPer plus Taq HiFi PCR mix (with blue dye) (Mei5 Biotechnology, Beijing). The identified positive clone samples were then sent to Sangon Biotech (Shanghai) for bidirectional sequencing.

### Bioinformatics analysis of *ShOMT3* gene sequence

Using DNAMAN software, the *ShOMT3* gene sequence was aligned and compared. The physicochemical properties of *ShOMT3* protein were analyzed using the ProtParam (Expasy - ProtParam tool) [41]. The conservative domains and superfamilies of the *ShOMT3* protein were predicted using the NCBI database (NCBI Conserved Domain Search (nih.gov)) [42]. SignalP 5.0 (SignalP 5.0 - DTU Health Tech - Bioinformatic Services) [43] was used to predict the presence of a signal peptide, while DeepTMHMM (DeepTMHMM 1.0 - DTU Health Tech - Bioinformatic Services) [44] was employed to predict transmembrane helices. SubLoc (Cell-PLoc 2.0 package (sjtu.edu.cn)) [45] was utilized to determine the subcellular localization of the protein. SOPMA (NPS@: SOPMA secondary structure prediction (ibcp.fr)) [46] was employed to predict and analyze the secondary structure of the protein, and Swiss-model (SWISS-MODEL Interactive Workspace (expasy.org)) [47] was used to predict and analyze its tertiary structure. Finally, a gene phylogenetic tree was constructed using the neighbor-joining method in MEGA5.0 [48].

## Results

### Cloning and sequence analysis of the *ShOMT3* Gene

**Fig 1:**
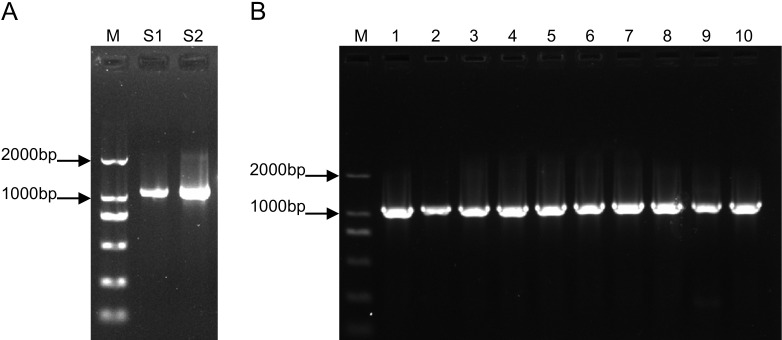
Cloning of *ShOMT3.* (A) PCR Amplification of *ShOMT3* in S1 and S2, (B)along with PCR Results from Bacterial Cultures of S1 [1–5] and S2 [6–10].

Using the cDNA templates reverse-transcribed from the total RNA of S1 and S2, PCR amplification products were detected by 1% agarose gel electrophoresis. The results indicated that *ShOMT3* was successfully cloned (Fig 1A). The CDS sequence of *ShOMT3* is 1119 bp long. Colony PCR was performed to detect the inserted fragment (Fig 1B), and positive clones were randomly selected for sequencing.Sequencing analysis results showed that the sequencing results of the *ShOMT3* gene fragments amplified from S1 and S2 were consistent with the *ShOMT3* gene sequence in the NCBI database (Genebank: MW531736.1).

Physical and Chemical Properties Analysis of *ShOMT3* Protein

The physicochemical properties of *ShOMT3* protein were analyzed using the online tool ProtParam (Expasy - ProtParam tool). The results showed that the gene is 1119 bp in length and encodes 372 amino acids. The predicted molecular formula of the protein is C1864H2912N472O541S23, with a molecular weight of 41.32784 kD and a theoretical isoelectric point (PI) of 5.27. ProtParam analysis indicated that leucine (Leu) is the most abundant amino acid, accounting for 11.0%, while tryptophan (Trp) is the least abundant, accounting for 1.3%. The protein does not contain pyrrolysine (Pyl) or selenocysteine (Sec). There are 31 positively charged residues (Arg+Lys) and 45 negatively charged residues (Asp+Glu). The instability index is 46.05, the aliphatic index is 93.58, and the grand average of hydropathicity (GRAVY) is 0.037.

Subcellular Localization, Transmembrane Domain Structure, and Signal Peptide Prediction of *ShOMT3* Protein

**Fig 2:**
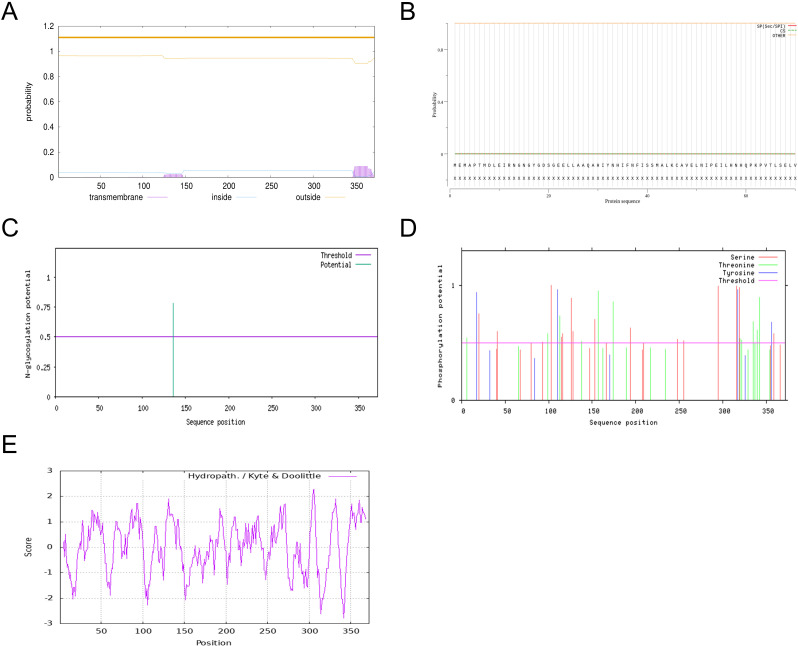
(A) Prediction of potential protein transmembrane, (B)protein signaling peptide, (C)glycosylation sites, (D)protein phosphorylation site and (E)hydrophilicity/hydrophobicity of *ShOMT3* protein.

Using the online analysis tool SubLoc (Cell-PLoc 2.0 package (sjtu.edu.cn)), the subcellular localization of the *ShOMT3* protein was predicted. The results showed that the *ShOMT3* protein is located in the cytoplasm. Additionally, the protein transmembrane structure was predicted using the online analysis tool DeepTMHMM (DeepTMHMM 1.0 - DTU Health Tech - Bioinformatic Services). The results (Fig 2A) indicated that the *ShOMT3* protein does not contain a transmembrane structure, with an expected value of 2.50036 for the number of transmembrane helices (more than 18 may contain transmembrane helices or signal peptides). Among the first 60 amino acids, the expected value for the number of transmembrane helices was only 0.01492. Furthermore, the N-terminus of the protein is located on the cytoplasmic side of the membrane with a total probability of 0.03634. In summary, the *ShOMT3* protein belongs to a non-transmembrane protein and is not localized to the membrane.

Using the online analysis tool Signal-P 5.0 (SignalP 5.0 - DTU Health Tech - Bioinformatic Services), the prediction of the signal peptide of the *ShOMT3* protein was carried out. The results (Fig 2B) indicated that the *ShOMT3* protein does not contain any signal peptides, making it a non-secreted protein. Additionally, using the NetNGlyc - 1.0 online analysis platform (NetNGlyc 1.0 - DTU Health Tech - Bioinformatic Services), the prediction of glycosylation modification sites on the *ShOMT3* protein was performed. The results (Fig 2C) indicated that the protein sequence contains only one glycosylation modification site at position 136. Furthermore, the analysis platform predicted that the ShOMT protein may not contain a signal peptide. Using the Netphos 3.1 server online analysis platform (NetPhos 3.1 - DTU Health Tech - Bioinformatic Services), the prediction of phosphorylation sites on the *ShOMT3* protein was carried out. The results showed that the protein contains 31 phosphorylation sites, including 16 serine (S) residues, 11 threonine (T) residues, and 4 tyrosine (Y) residues (Fig 2D). Additionally, using the Expasy-protscale online analysis platform (Expasy - ProtScale), the analysis of the hydrophilicity/hydrophobicity of the *ShOMT3* protein was performed. The results (Fig 2E) demonstrated that the amino acid scale values of *ShOMT3* ranged from a maximum of 2.289 to a minimum of -2.789, indicating an overall insignificant difference. Therefore, the *ShOMT3* protein does not exhibit a pronounced hydrophobicity or hydrophilicity.

### Domain analysis and structural prediction of *ShOMT3* protein (including secondary and tertiary structures)

**Fig 3:**
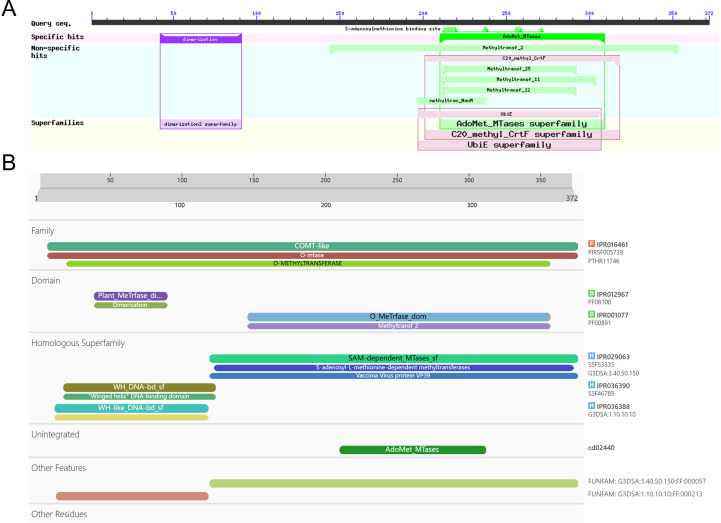
Analysis of conserved domain by CDD Searcher (A) and InterPro (B).

By querying the InterPro (InterPro (ebi.ac.uk))and NCBI databases using CDD Searcher (NCBI Conserved Domain Search (nih.gov)), the conservative domains of the *ShOMT3* protein were identified. The results (Fig 3) indicated that the *ShOMT3* protein contains two conservative domains: an S-adenosylmethionine binding site [chemical binding site] encoded by amino acids 144–353 and a pfam08100 domain encoded by amino acids 42–91. These domains belong to the AdoMet_MTases super family and Dimerisation domain super family, respectively. Additionally, using InterProScan (InterProScan - InterPro (ebi.ac.uk)), the *ShOMT3* protein was screened and analyzed, and it was determined that the protein belongs to the IPR016461 family, which is a type of SAM-dependent methyltransferase (O-methyltransferase COMT-type). The amino acid residues at positions 214, 237, 257, 258, and 271 are likely to form the binding site of *ShOMT3* with S-adenosyl-L-methionine, responsible for catalyzing the reaction between S-adenosyl-L-methionine and (-)-pluviatolide to yield (-)-bursehernin and S-adenosyl-L-homocysteine. Furthermore, the amino acid residues at positions 275, 306, and 338 are potentially the active sites of *ShOMT3*, with the residue at position 275 being predicted to serve as the proton acceptor.

**Fig 4:**
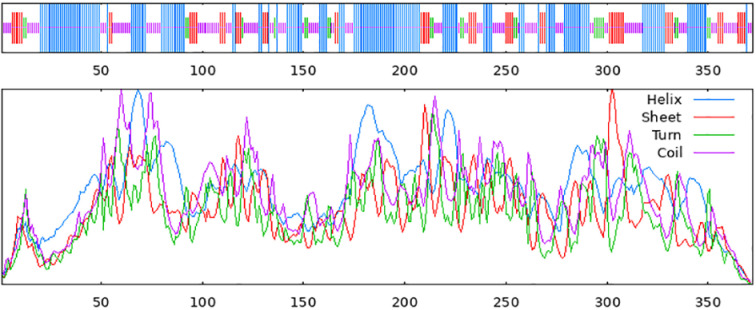
Secondary structure prediction of *ShOMT3* protein.

**Fig 5:**
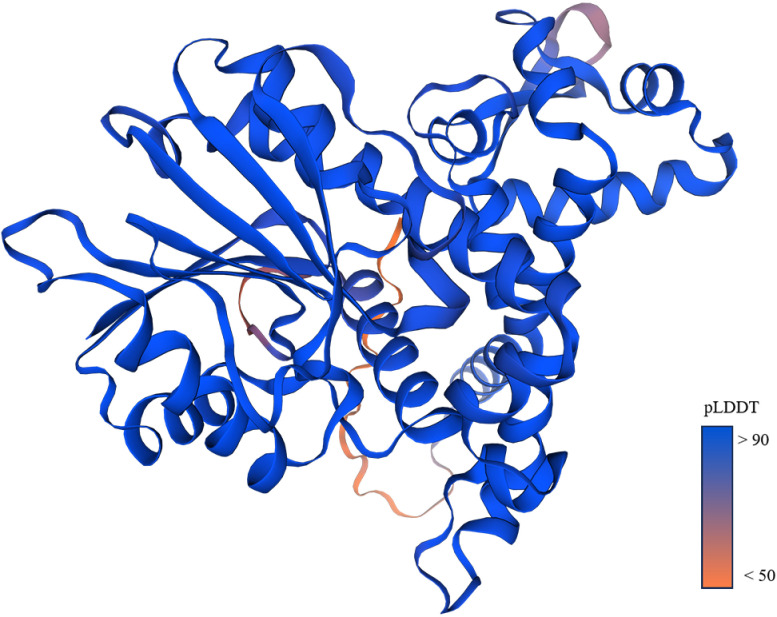
Three-dimensional structure prediction. The different coloring represents Per-residue model confidence score (pLDDT) between 0 and 100. Some regions with low pLDDT may be unstructured in isolation.

Using the SOPMA online analysis software (NPS@: SOPMA secondary structure prediction (ibcp.fr)), a prediction analysis of the secondary structure of the *ShOMT3* protein was carried out. The results (Fig 4) indicate that, throughout the *ShOMT3* protein, alpha-helices account for 43.82%, random coils contribute 33.60%, extended strands make up 15.86%, and beta-turns represent 6.72%. Therefore, the *ShOMT3* protein is composed of four secondary structural states. Based on the automated mode of homology modeling using the Swiss-model (SWISS-MODEL Interactive Workspace (expasy.org), the *ShOMT3* protein was structurally predicted using Pluviatolide O-methyltransferase A0A0N9HMN6.1.A as a structural model. The results (Fig 5) indicate a Seq Identity of 99.46%.

### The homology analysis and phylogenetic analysis of *ShOMT3* protein

**Fig 6:**
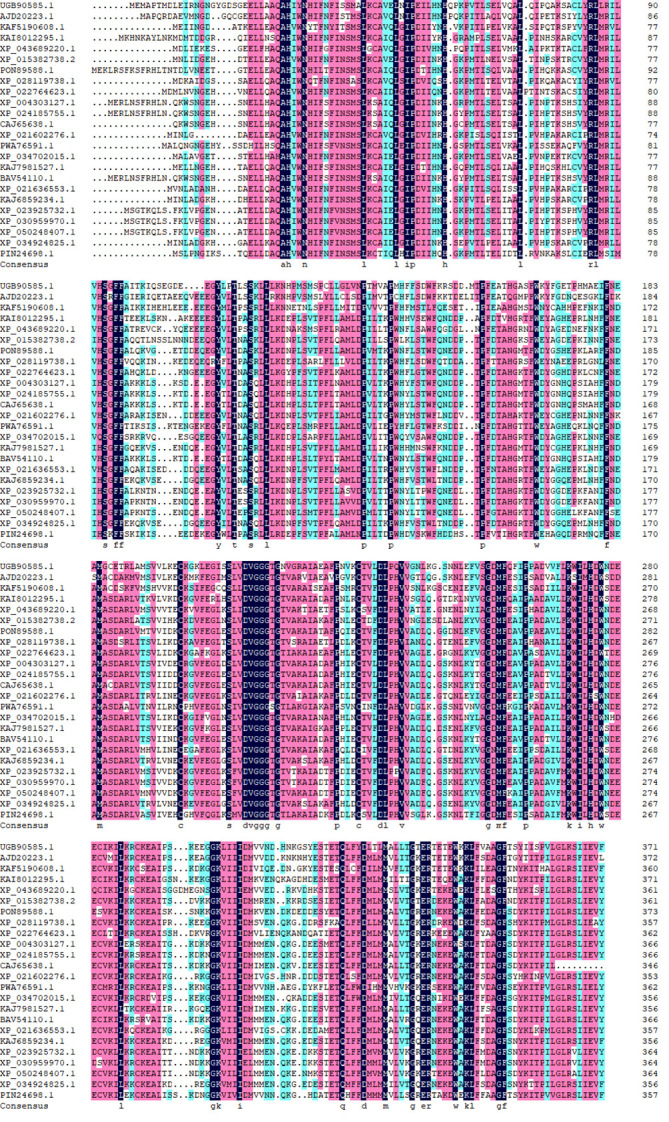
Comparison of *ShOMT3* amino acid sequences between *S. hexandrum* and other plants.

By utilizing the Blast analysis tool on the NCBI website, a search and alignment of the *ShOMT3* amino acid sequence was conducted. Subsequently, based on the DNAMAN software, homology analysis was performed on the aligned amino acid sequences. Homology analysis allows for the comparison of more than 20 protein sequences that belong to the same family, are evolutionarily related, or have structurally similar features. The results (Fig 6) indicate that the amino acid sequence of *ShOMT3* protein (Genebank: UGB90585.1) has the highest similarity to that of the 6-O-methyltransferase of *P. peltatum* (AJD20223.1), with a homology rate of 67.47%. This indicates that these two sequences are evolutionarily related and have the closest genetic relationship. This result is consistent with the classification of *P. peltatum* and *S. hexandrum* in plant taxonomy.

**Fig 7:**
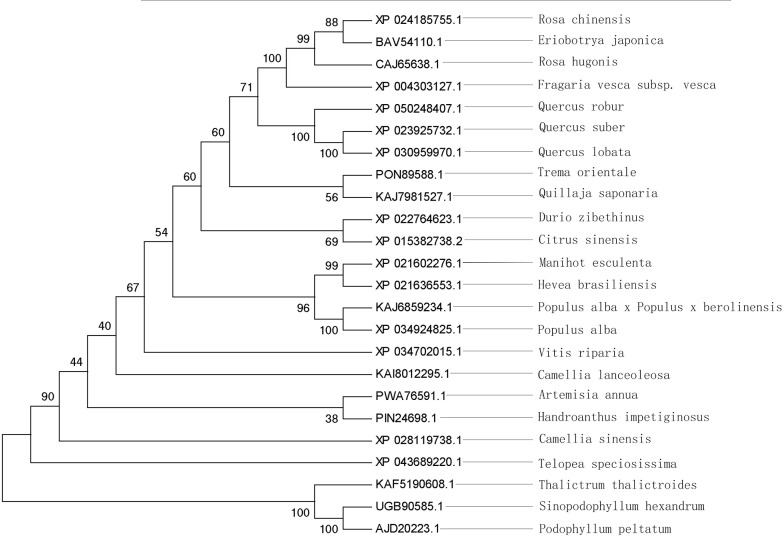
Phylogenic tree analysis of different plants on *ShOMT3* amino acid sequence.

Using the neighbor-joining method and MEGA 5.05 software, a molecular phylogenetic tree was constructed for the *ShOMT3* gene sequence. The results (Fig 7) indicate that UGB90585.1 first forms a branch with AJD20223.1, indicating that *P. peltatum* has the highest homology with *S. hexandrum*. This is followed by Thalictrum minus and Anemone.

## Discussion

Podophyllotoxin and its derivatives are important components in anti-cancer drugs, and have significant potential for medical applications [12]. It is necessary to explore the biosynthetic pathway of podophyllotoxin and understand the biological information of the key regulatory genes involved in its biosynthesis process to facilitate the improvement of the current production method of podophyllotoxin and address the supply shortage faced in the market. In recent years, research on the genetic mining of regulatory enzymes in the podophyllotoxin biosynthetic pathway of *S. hexandrum* has become increasingly in-depth [49–51]. However, to date, the majority of research has centered on the earlier discovered genes related to the biosynthetic pathway of the precursor compound, pinobanksin, such as *ShPAL*, *Sh4CL*, and *ShCAD* [34,52,53]. OMTs serve as a crucial regulatory enzyme in the phenylpropanoid metabolic pathway, and several OMT enzyme have been identified in the podophyllotoxin biosynthetic pathway, including ShCOMT, ShCCoMT, ShOMT1, and *ShOMT3* [54–56]. ShCOMT is responsible for regulating the conversion of caffeic acid to ferulic acid, while ShCCoMT is responsible for converting caffeoyl-CoA to feruloyl-CoA [57,58]. These two OMT enzyme play an important regulatory role in the synthesis of podophyllotoxin precursor compound pinobanksin, and have been isolated in multiple plants [59,60]. However, the regulatory role of *ShOMT3* and ShOMT1 in the podophyllotoxin biosynthetic process has only been discovered in recent years, and related studies have not yet been conducted in-depth.

In this study, we attempted to clone the *ShOMT3* gene of S1 and S2. The prediction of the physicochemical properties and hydrophobicity of the *ShOMT3* protein indicates that it is a hydrophobic unstable protein. The subcellular localization analysis of the *ShOMT3* protein suggests that it may be located in the cytoplasm. The prediction of transmembrane structure and signal peptide analysis indicates that although a few amino acid residues in the *ShOMT3* protein are predicted to contain transmembrane helices, the *ShOMT3* protein does not have a transmembrane region or a signal peptide. Proteins without signal peptides are unlikely to be exposed to N-glycosylation mechanisms, so even if the ShOMT protein contains potential glycosylation motifs, it may not be glycosylated (in vivo). This also suggests that the *ShOMT3* protein is a non-secreted protein and is not located on membranes, but may be located in the cytoplasm or organelles, consistent with the subcellular localization analysis results and consistent with the analysis of OMT3 proteins in *Vitis vinifera* [61].

The prediction of the *ShOMT3* protein structure domain shows that the *ShOMT3* enzyme belongs to the IPR016461 family, which is a type of O-methyltransferase COMT-type (IPR016461). The *ShOMT3* protein contains AdoMet_MTases superfamily domain type I and dimerization domain, which is similar to COMT [62]. The AdoMet-MTases belong to the S-adenosylmethionine-dependent methyltransferase family, indicating that the *ShOMT3* protein uses S-adenosyl-L-methionine (SAM or AdoMet) as the methyltransferase substrate to produce S-adenosyl-L-homocysteine (AdoHcy) as the product [26]. The AdoMet-MTases can be divided into at least five structurally distinct families, with the I class being the largest and most diverse [63]. Within this class, enzymes can be further classified based on their substrate specificity (small molecules, lipids, nucleic acids, etc.) and the type of methyltransferase target atom (nitrogen, oxygen, carbon, sulfur, etc.) [24]. The dimerization domain has been demonstrated to mediate the dimerization of various plant OMTs, typically located at the N-terminal of these proteins [64,65]. This domain plays a crucial role in the catalytic activity of OMTs [66]. Based on the secondary and tertiary structure prediction results, it can be speculated that the *ShOMT3* protein may function as a monomer in catalysis, consistent with existing research on other type I OMTs [67]. The amino acid sequence of the *ShOMT3* protein (UGB5098585.1) exhibits the highest homology with that of *P. peltatum* (AJD20223.1) in the phylogenetic tree, consistent with the trend of biological evolution. This result supports the positioning of the *ShOMT3* gene in biological evolution to some extent. The construction of the molecular phylogenetic tree indicates that it fully reflects the relationship of species’ affinity, and OMTs from different sources can be classified into different taxonomic groups, without deviation from the biological classification. It is consistent with the traditional classification. Additionally, these results indirectly indicate the accuracy of the *ShOMT3* gene sequencing and bioinformatics analysis results.

This study aims to understand the structure and biological characteristics of *ShOMT3* by cloning the coding sequence (CDS) region and conducting bioinformatics analysis. This will provide a theoretical foundation for further exploring the functional and enzymatic properties of *ShOMT3*. The analysis of the basic biological characteristics of *ShOMT3* provides valuable genetic resources and theoretical support for further exploring the molecular regulatory mechanism of podophyllotoxin biosynthesis in *S. hexandrum*. This study lays a foundation for better utilization and development of the medicinal and economic value of *S. hexandrum.*

## Conclusion

In this study, the key regulatory gene *ShOMT3* for podophyllotoxin biosynthesis was successfully cloned from S1 (Gansu) and S2 (Shaanxi). A comprehensive bioinformatics analysis was also conducted. In the phylogenetic analysis, the *ShOMT3* gene showed the closest genetic relationship with *P. peltatum*, with a high homology rate of approximately 67.47%. This is consistent with the plant classification results for *P. peltatum* and *S. hexandrum*. The analysis of the basic biological characteristics of the *ShOMT3* gene provides valuable genetic resources and theoretical support for further exploring the molecular regulatory mechanism of podophyllotoxin biosynthesis in *S. hexandrum*. This study lays a foundation for better utilization and development of the medicinal and economic value of *S. hexandrum.*

## Supporting information

S1_raw_images Raw images(pdf). (PDF)
