# Supplementary figures and images for: Cloning and bioinformatics analysis of key gene ShOMT3 of podophyllotoxin biosynthesis pathway in Sinopodophyllum hexandrum
Source: PLoS One. 2025 Feb 14;20(2):e0314919. doi: 10.1371/journal.pone.0314919 (PMC11828346; doi:10.1371/journal.pone.0314919)

A

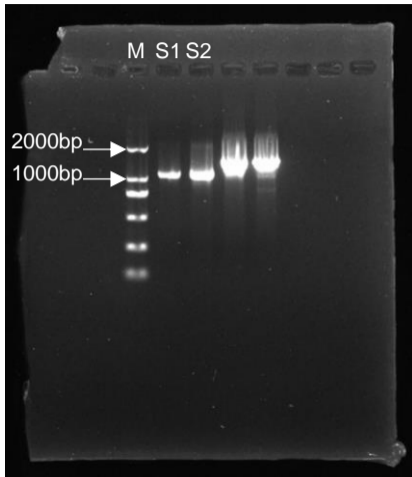

B

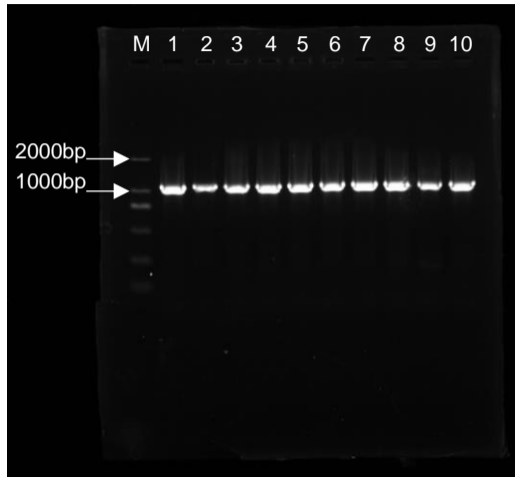

Supplement: S1_raw_images — (PDF) [file pone.0314919.s001.pdf]
